# Cognitive–Linguistic and Constructivist Mnemonic Triggers in Teaching Based on Jerome Bruner’s Thinking

**DOI:** 10.3389/fpsyg.2018.02543

**Published:** 2018-12-12

**Authors:** Jari Metsämuuronen, Pekka Räsänen

**Affiliations:** ^1^Department of Pedagogy, NLA University College, Bergen, Norway; ^2^Niilo Mäki Institute, Jyväskylä, Finland

**Keywords:** Jerome Bruner, constructivist learning theories, cognitive psychology, educational psychology, mnemonics, mnemonic triggers, teaching

## Abstract

Effective teachers use mnemonic tools or mnemonic triggers to improve the students’ retention of the study material. This article discusses mnemonic triggers from a theoretical viewpoint based on Jerome S. Bruner’s writings. Fifty small linguistic–cognitive, constructive-, rhetorical-, and phonological–mnemonic triggers are detected. These triggers may become supporting elements for our memory system when we are “constructing the realities” in a Brunerian sense when we are ordering, differentiating, comparing, and handling information, stories and experiences in our mind. Many of these are small, hidden linguistic elements in speech. This article discusses their usage in the educational talk and textbooks.

## How to Teach the Material So That It Could Be Remembered the Most Effective Way?

Let us visualize a simple test of listening to a list of 31 words ordered in an alphabetical order and trying to remember those by heart:

A, And, Are, Ask, Because, Carefully, Concepts, Examination, Final, I, Important, In, Listen, Now, Of, Of, Psychometrical, Referring, Reliability, Terms, Test, The, The, The, These, To, Trustworthiness, Ultimately, Validity, When, Will

Some other rationale such as the length of the words could be used also in ordering the list. Even if the test-takers would not be familiar with concepts in the list, it is obvious that, after listening to the material repeatedly, they would learn the words by heart without a problem within a couple of tens of minutes—maybe even in 10 min. Some of the test-takers would need somewhat more time with the task than some others would do but, anyhow, all could solve the task [see the classical experiments of learning and memorizing the lists of foreign words by Tulving (1967) and Roediger and Karpicke, 2006a,b; Karpicke and Roediger, 2008]. That is not a problem.

The task would be much easier and faster if we organized the list of words in the following sequence:

Now, listen carefully because I will ask these concepts in the final examination. The terms of reliability and validity are ultimately important when referring to the trustworthiness of a psychometrical test.

We may ask why we would remember the words better, faster, and prolonged way when the data was given in the latter order instead of the former order. O’Keefe and Nadel (1998), pp. 388–389) propose when the information is categorized either in the verbal or visual form, it reduces the amount of information that needs to be retrieved. They assume a kind of semantic map in the brain (specifically in hippocampus) (*ibid*. p. 410). This article discusses the matter from mnemonic triggers viewpoint and proposes a hypothesis related to the example above: *we use hidden or obvious linguistic–cognitive, constructive-, rhetorical-, and phonological mnemonic triggers to guide the attention, and to enhance the encoding, and recall of the material to be learnt*. The questions discussed in this article are what those triggers are and how to detect those. The focus is on classifying the triggers based on the relevant research literature, and to form a theoretical framework for the further studies and practical use.

From the educational psychology viewpoint, this brings us to an essential practical question: what kind of teaching talk or study material is effective and why? In this article, the effectiveness is narrowed down to retention and retrieval—such teaching or educational material is effective which leaves a measurable change in the memory or in the behavior of a student. This measurable change, or “a memory” may be detected physically from the brain^1^ and can be recalled or observed as a change in the behavior of the learner. Here, the concept of “teacher” and “teaching” is enlarged to cover not only the instructor as a human being, but also the consciously organized teaching material such as books, articles, or lecture notes meant to be read and learnt without the human instructor as well as, in the near future, virtual teachers run by artificial intelligence. The writer of a textbook or an article in a compendium for students *is* a teacher though not necessarily physically present. This brings us close to the idea of two types of teaching discussed by Biesta (2013, 2016): “learning from” and “being taught by” a teacher. The first means that a teacher is a *resource* for the student and the latter implies a “teaching” teacher. Both are relevant aspects of being a teacher.

An effective teacher uses such methods deliberately that are aimed to improve the retention and recall of the study material. We call these methods mnemonic tools (e.g., Bafile, 2005), mnemonic devices (e.g., Sökmen, 1997; Haydon et al., 2017, pp. 240–241), mnemonic strategies (e.g., Mastropieri et al., 1992; Mastropieri and Scruggs, 1998), mnemonic instructions (e.g., Mastropieri and Scruggs, 1989, 1991; Lubin and Polloway, 2016), or mnemonic triggers (e.g., Metsämuuronen, 2010).^2^ These mnemonic triggers are the subject of this article. Mnemonics and mnemonic instructions have been studied widely specifically within the special education (see literature in Mastropieri and Scruggs, 1998; Lubin and Polloway, 2016). This literature is not reviewed here as the focus is on Bruner’s ideas and possible *new* mnemonic triggers found in his writings. However, as a reasonable outcome of the literature, Mastropieri and Scruggs (1998), p. 1) note: “We recommend mnemonic strategies for only one reason: Over and over again, they have been proven to be extremely effective in helping people remember things.”

Some elementary concepts and theories of constructive psychology are discussed in Section “Constructivism and Cognitive Psychology in the Contemporary Educational Discourse” and of cognitive psychology in Section “Memory and Cognitive Models.” Brunner’s legacy in the educational psychology and his ideas and the basic mnemonic triggers are discussed in Section “Bruner and Mnemonic Triggers.”

## Constructivism and Cognitive Psychology in the Contemporary Educational Discourse

Regardless some few critical voices^3^, constructivist learning theories and constructivism have superseded, more or less, the naturalistic approaches, such as the behaviorist and cognitivists learning theories, in the contemporary educational practices, discourse, and language. There are several reasons for this. One is that constructivism justifies students’ being active learners instead of passive receivers. Another is that the development of higher-level thinking, including the complex use of language, is very difficult to explain from the behaviorist and cognitivist approaches. We can easily find several more reasons. Despite the possible challenges^4^ in the epistemological base of the constructivism (see Puolimatka, 1999, 2003; Nodding, 2016, p. 122), the practical educators have found constructivist learning theories helpful in their work and many researchers from deviating aspects have used these as relevant background theories for their studies. Some of the recent publications with constructivist learning theories as the theoretical framework have tackled the use of technology (Alabdulaziz and Higgins, 2017; Harasim, 2017), business ethics (Lämsä et al., 2017), professional development (Shore and Morris, 2016), curriculum studies (van Bommel et al., 2015), higher education (Bhola and Parchoma, 2015), augmented reality teaching (Dunleavy and Dede, 2014), experiential learning (Kolb, 2014), and linguistics (Jiang and Perkins, 2013)—just to mention a few. Though there are different theories and practical solutions in constructivism in the educational realm these share the general humanistic basic tenet that “*people construct their own understanding and knowledge of the world through experiencing the world, and reflecting on those experiences*,” as expressed by Harasim (2017, p. 62). Duffy and Cunningham (1996, p. 177) put it as “*learning is an active process of constructing rather than acquiring knowledge*.” Both of these originate from Bruner (1961).

Constructivist learning theories are one branch of the movement of cognitive sciences strongly affected by Jerome S. Bruner’s works from the 1950s on (see Bruner and Goodnow, 1986). While the constructivist learning theories rose from the humanistic tradition (from Piaget and Bruner) and social cultural tradition (from Vykotsky, 1925), in the same wave of cognitive sciences, the cognitive psychology developed from Miller (1956) and Broadbent (1958) from the tradition of natural sciences, and cognitive linguistics from Chomsky (1957) from the tradition of linguistics. All these traditions have tried to explain the “mind,” or mental processes, in a human—constructivists from the humanistic viewpoint, cognitive linguistics from the semantics viewpoint, and psycholinguistics or cognitive psychologists from the neural viewpoint. They all share the basic tenet that at least part of the human linguistic ability is innate, and that language is embedded in the overall cognitive capacities of man (Geeraerts, 1995, p. 111; Taylor, 1984, p. 223). Also, the basic theories (or at least their foundations) of the storage and retrieval, or memory and retention, of linguistic data are largely shared. Though having been criticized by constructive psychologists (e.g., Bruner, 1985, p. 31), cultural psychologists (e.g., Schweder, 1991, p. 73), educational psychologists (e.g., Säljö,, 2000, p. 56), and philosophers (e.g., Taylor, 1985) cognitive neuroscience has opened promising doors to understanding how the human mind actually works at the neural level. Taylor (1984, p. 223) reminds us that also the constructivists (should) think that there has to be a common biogenetical and personal developmental ground in our minds—otherwise it would be impossible to communicate with each other. According to Taylor (1984, p. 212), in Bruner’s thinking, the language skill, that is, our thinking, is based on biological factors but this biological capacity requires cultural expression. Bruner’s thinking seems to be a kind of link among the humanistic constructivism, naturalistic cognitive neurosciences, and cognitive linguistic.

Bruner’s role in the development of the modern educational thinking is important. This article combines his essential ideas of cognitive processes related to learning and tries to find a practical theory for the empirical works of the essential cognitive–linguistic and constructive triggers that are elementary for constructing the “possible worlds” (Bruner, 1986). This article is mainly theoretical, and the triggers are discussed in the light of Bruner’s writings.

## Memory and Cognitive Models

According to the widely accepted Atkinson and Shiffrin (1968) model, the key processes of learning and memory are multi-staged. When new information is taken in, it is in some ways manipulated before it is stored. This stage theory describes three types of memory: sensory memory, working memory, and long-term memory. The short-term or working memory refers to our capacity to hold a small amount of information in an active state while doing a task (originally proposed by Miller, 1956; see also Baddeley, 1997, 2003; Miyake and Shah, 1999). The contents of the long-term storage and retrieval from it are strongly dependent how information is processed at the earlier stages.

The basic theories of human mind claim that the human long-term memory can be divided into two main categories: declarative memory and procedural (or non-declarative) memory (e.g., Squire, 2009; Eysenck and Keane, 2010). Declarative memory concerns things that can be brought to mind and declared, that is, facts that can be explicitly stated. Procedural memory, on the other hand, stores the motor and cognitive skills and habits and its contents cannot be put into words (Poldrack and Packard, 2003; Ullman, 2004; Squire, 2009). Declarative memory can be further divided into semantic and episodic (or narrative) memory (e.g., Tulving, 1983; Bruner, 1986, 1990a). Episodic memory consists of a store of the memories of personal events and actions. The units of episodic memory are events and episodes. Schacter et al. (2007) describe an additional role for the episodic memory system. Episodic memory is by large constructive, and therefore it allows us not only to think about the past experiences, but also to build mental simulations of the imagined future. In this sense, episodic memory may serve in a large role, e.g., in decision-making, creativity and problem-solving (Madore et al., 2017).

Semantic memory is connected to the knowledge concerning the world—it is independent of the identity of the person and of personal history (Tulving, 1983, p. 9). The units of semantic memory are facts and concepts. The content of semantic memory is something the individual *knows* whereas the content of episodic memory is something the individual *remembers*. The semantic memory is organized in concepts and episodic memory is organized in time.

Memory depends on attention; attention and memory cannot operate without each other (Chun and Turk-Browne, 2007). Attention and its connection to brain activities and memory is widely studied (see, practical studies, for example, by Simola et al., 2014; Moisala, 2017; Salo et al., 2017; Rämä et al., 2018), and only some basic ideas are raised here to connect the mnemonic triggers to attention. Chun and Turk-Browne (2007) suggest that, first, memory has a limited capacity, and, hence, attention determines what will be encoded and, second, memory from past experiences guides what should be attended. Cowan (1988, 1998) have proposed a model how the attention and memory are linked to each other. According to Cowan’s model, although relatively unprocessed elements of long-term memory can be automatically activated, new associations between items, and between each item and its context, may be set up only in the focus of attention. Information that is temporarily in or near conscious awareness is in the focus of attention. Some attention is probably needed to perceive items adequately. Beyond that, one can distinguish between memory with less versus more attention devoted at the time of encoding.

The memory retrieval is determined by the conditions of acquisition or encoding and the relation between encoding and retrieval operations. The more meaningful the analyses of stimuli at hand are, the higher the levels of subsequent retention will be (Craik and Lockhart, 1972; Craik, 2016). While this levels-of-processing effect has been mostly studied in the context of verbal information, in their recent study, Baddeley and Hitch (2017) showed the similar mechanisms can be found in retention of visual information.

Cognitive models assume that the retention and retrieval of memory can be explained by co-operation between working memory and long-term memory. Working memory refers to the temporary retention of information that was just experienced or just retrieved from long-term memory. It is short-lived but can be stored for longer periods of time through active maintenance or rehearsal strategies. Even though multiple factors are connected to better results in memory tasks, declarative memories are best established by using active recall combined with mnemonics and spaced repetition^5^ (Tulving and Schacter, 1990; Baddeley, 1997). The wealth of studies has shown their benefits in designing education and pedagogies to boost long-term retention (see recent reviews, e.g., Toppino and Gerbier, 2014; Larsen, 2018). In the models of Cowan (1998, 2017) and Anderson (1983) the working memory is not considered as a separate storage buffer but functions via different levels of activation of the long-term memory storage that is distributed in various areas of the brain. In this way, the memory retrieval plays an important role in the functioning of the working memory. Likewise, these models connect the attention-directing part of the working memory, “the central executive” (Baddeley, 1997) to long-term storage retrieval.

A basic doctrine of human learning and memory research is that repetition of material improves its retention (see Tulving, 1967). This tenet was challenged by Karpicke and Roediger (2008), Roediger and Karpicke (2006a,b) and earlier by Tulving (1967). Their experiments showed that delayed recall is optimized, not with repeated *studying* sessions, but with repeated *testing* sessions. Metsämuuronen (2013); also, Metsämuuronen and Mattsson (2013) shows practical results supporting this theoretical result. The result was re-interpreted by Lasry et al. (2008). They hypothesized that repeated testing might lead to *multiple traces* to the memory, which facilitate recall, and suggested that the new interpretation would lead to a new framework for explaining the effectiveness of frequent in-class assessments in pedagogies such as Peer Instruction. Alternatively, these could be organized by using feedback via cues to guide the task process (Hattie and Timperley, 2007) often with the aid of learning technologies (e.g., Van der Kleij et al., 2015).

## Bruner and Mnemonic Triggers

### Bruner’s General Role in the Educational Psychology

Jerome S. Bruner (1915–2016) is one of the key figures of the modern constructivist theories in education along with the Swiss psychologist Jean Piaget (1896–1980) and Russian psychologist Lev Vygotsky (1896–1934).^6^ Piaget developed the theory of the thinking processes of a child from his early studies from 1926 onward (Piaget, 1929; see the literature in Beard, 2007) and these had a significant role in the development of *cognitive* constructivist learning theories. Vygotsky developed the *social* constructivist learning theories in his *Psychology of Art* (1925) and later works.

Bruner was born blind and it may have had an effect on his later career. He himself noted that, during the first two blind years, he had constructed a visual world in his mind (Greenfield, 2016). Hence, he had a strong intuition that perception is not just controlled by senses but also by mind. His early study *A Study of Thinking* (Bruner et al., 1956), played a pivotal role in the cognitive revolution that is now called the cognitive sciences. Later, this thinking manifested as cognitive psychology^7^ and as constructivist learning theories or constructivism in education in a wide sense (see Harasim, 2017, p. 62). *The Process of Education* (Bruner, 1960) brought the cognitive revolution to educational discussion. Bruner proposed the idea of a spiral curriculum where a more complex idea can be thought at a simplified level first and at a more complex level later. *Act of Discovery* (Bruner, 1961) led to the concept of “discovery learning.” Bruner proposed that learners construct their own knowledge by organizing and categorizing information using a coding system. The most effective way to develop a coding system is to discover it rather than being told it by the teacher. In *Toward a Theory of Instruction* (Bruner, 1966) and *Studies in Cognitive Growth* (Bruner et al., 1966) he proposed, on the basis of his earlier study (Bruner, 1964), three modes of representation, or as interpreted today, three levels of learning: enactive representation (action-based), iconic representation (image-based), and symbolic representation (language-based). His *Actual Minds, Possible Worlds* (Bruner, 1986) brought the narratives in the center in creating different worlds in the minds. It is one of the most cited academic books in history (see Greenfield, 2016).^8^ In *Acts of Meaning* (Bruner, 1990b), Bruner proposed that human behavior is ultimately unintelligible without reference to such mental concepts as intentions and goals, and, as nuanced by Rendall (1991), suggests that the fear of a debilitating relativism results from ignoring the social context within which the acts of meaning take place. *The Culture of Education* (Bruner, 1996) is a collection of essays, addresses, and lectures by him about cultural psychology. This book has made some scholars think that Bruner changed his educational thinking in his later years (see discussion in Takaya, 2008).

Bruner was an ultimate optimist for education. He proposed: “We begin with the hypothesis that any subject can be taught effectively in some intellectually honest form to any child at any stage of development” (Bruner, 1960, p. 33). In this matter, he opposed Piaget who thought that there are certain fixed steps for learning dependent on the development of the child. Considering his tremendous influence in the educational practices as well as works and theoretical pondering of scholars in various fields, it is no wonder why Haggbloom et al. (2002) ranked Bruner as one of the most cited^9^ psychologists of the 20th century. His influence reaches much farther than the academic citations psychological journals or psychological textbooks: his ideas have changed the educational thinking and systems in many countries.

### Bruner and Cognitive–Linguistic Mnemonic Triggers

According to Bruner (1983, p. 164), Bruner (1986, p. 114) we use language to communicate, to differentiate between and to order things, and to construct realities. From this point of view, such cognitive–linguistic operations as *connecting*, *differentiating*, *comparing*, and *ordering* things as well as *constructing realities* can be thought to be universal ways to save and handle information, stories, and experiences in our brains. The challenge concerning the cognitive–linguistic operations is that, though they are universal, they are language- and syntax specific. Here, the English words are used as examples.

Two simple linguistic triggers for connecting things by using **doubles** in English are “and” or “or” (“X *and* Y”; “X *or* Y”). We can connect a whole variety of things, for example, “black *and* white.” In this example, *two colors* are connected (connecting things from the *same* category, i.e., color), *separate* colors are connected (connecting things from *different* categories, i.e., black and white), the *order* of the colors is connected (connecting things from ordered shades, i.e., from the darkest to the lightest), and metaphorically *opposite* colors are connected (connecting two extremes). It depends on the situation and the intellectual level of the listeners as to how they understand and interpret the phrase. This expression of doubles is quite similar to another indicator of oral transmission, the expression of three things, triple repetitions, which is a common mnemonic method in narratives (see Section “Bruner and Narrative Mode of Thinking—Metaphors, Similes, Narratives, and Triple Repetitions as Mnemonic Triggers”).

Three simple linguistic triggers for **comparing** and **differentiating** things in English are “like,” “as,” “or,” and the more complicated “but”: (“X is [like] A *but* Y is [like] B”). In a positive expression, the triggers “like” and “as” are used for a simile^10^ and, in a negative expression, for discrimination. The trigger “but” may differentiate things on two levels: separating two things from each other (nominal discrimination) and separating *opposite* things from each other (ordinal discrimination). Difference can be stated also by using **strict differentiating** with triggers “to make a difference,” “do not,” or “separate from” (“*separate* X from Y!” or “*do not do/be* X”). Comparison can be made also by using **strict comparison** like in “compare” or “the same way”: (“*compare* X with Y” or “*in the same way*, X is Y”).

A simple way to *create order* in concepts and things is to separate **nominal** **counterparts**: “head – toe” or “hands – feet” without specific reference to the order. We can also use **nominal order** with explicit or implicit order as in “small – big” or “weak – strong” or use explicit **comparative order** such as “smaller – bigger [than]” or “weaker – stronger [than]” or **superlative order** such as “the smallest – the biggest” or “the weakest – the strongest” or “A – Z.” Yet another way is to use general expressions of (ultimate) **extremes** such as “all,” “always,” “never,” or “in the end” and “finally.”

The last set of cognitive triggers addressed here briefly are the *linguistic* triggers for **constructing knowledge**—the *narrative* and *logical* triggers are handled in Sections “Bruner and Narrative Mode of Thinking—Metaphors, Similes, Narratives, and Triple Repetitions as Mnemonic Triggers” and “Bruner and Logical–Scientific Mode of Thinking—Logical Mnemonic Triggers.” A simple set of linguistic triggers for constructing knowledge are triggers for a light **argument** and **conclusion** such as “because of,” “for,” “so,” “thus,” “then,” “hence,” and “therefore” (“X is Y *because of* B” or “*Hence*, X is Y”). A more complicated way to construct realities is to **condition** something by the positive trigger “if” (“*if* X then Y”) or negative “unless” (“*unless* X, no Y”). We may find many more triggers of this kind. Some profounder logical triggers are handled with the logical–scientific mode in Section “Bruner and Logical–Scientific Mode of Thinking—Logical Mnemonic Triggers.”

The relevance of these triggers from the contemporary thinking of memory viewpoint is that these triggers make *meaning* to the stimulus. As known from the studies of Craik (2017) and Baddeley and Hitch (2017), the more meaningful the analyses of stimuli at hand are, the higher the levels of subsequent retention will be (see Section “Memory and Cognitive Models”). These triggers may also serve in packing the load of information; while using these kinds of connectors, the amount of information that needs to be retrieved may be reduced (see O’Keefe and Nadel, 1998, 388–389).

### Bruner and Constructivist Mnemonic Triggers

#### Building on What Is Already Known

The basic idea of the constructivist learning paradigm is that learning is an active, social process in which a student constructs new ideas or concepts based on his/her current knowledge (Bruner, 1961). One of the basic principles of constructivist education is that learning reinforces itself in a **spiral way**; new things are learned by building on the previous experiences (Bruner, 1960, p. 52). Bruner (1961) proposes that learners construct their own knowledge by organizing and categorizing information using a coding system which should be discovered rather than being told by the teacher.

Two kinds of references can be separated concerning previous experiences: first, **reference to something which is already known** in a general sense (“As you [already] know…”) or **reference to generally known common concepts** from everyday life (“You have three *apples* and give one away. How many *apples* are left?”). Another related mnemonic trigger is the **structural repetition^11^**: the teacher organizes the teaching or study material in such a way that repetition enhances the retention. In structural repetition, the same topic, word, concept or idea is repeated in the same or slightly modified way within the same teaching session or material package. Naturally, the teacher can use repetition also—**teaching the same topic several times**.

The relevance of these triggers from the contemporary thinking of memory viewpoint is that repeating the material is an effective device for remembering (e.g., Tulving, 1967; Baddeley, 1997) though not as effective as the **repeated** **testing** of the material studied (Tulving, 1967; Karpicke and Roediger, 2008; Metsämuuronen, 2013; Metsämuuronen and Mattsson, 2013). This may lead to multiple traces to the memory (Lasry et al., 2008); in the modern teaching processes these multiple traces can be enhanced by using feedback via cues during the task process and specifically with the aid of learning technologies (Hattie and Timperley, 2007; Van der Kleij et al., 2015).

#### Bruner and Narrative Mode of Thinking—Metaphors, Similes, Narratives, and Triple Repetitions as Mnemonic Triggers

According to Bruner (1986, p. 11), humans have two cognitive modes of thinking: logical–scientific mode and narrative mode (also see Section “Constructivism and Cognitive Psychology in the Contemporary Educational Discourse”). With both these modes, individual experiences are organized and ordered, and given meaning, and problem solving is explained (Bruner, 1986, p. 11; Bruner, 1996, pp. 39, 130). This section focuses on the narrative mode and the next section on the logical–scientific mode.

With narrative thinking, we can explain human behavior and psychic reality—we are willing to create connections between different facts. The narrative mode is focused in the affective and functional structures of teaching. In this mode, such elements as intentions, goals, subjective experiences, and the characteristics of individual are in focus (Bruner, 1986, p. 50; Bruner, 1990b, p. 710). This comes close the rhetorical elements in teaching—especially that of *pathos* (see Section “Bruner, Aristotelian Rhetoric and Mnemonic Triggers”). Narrative thinking is based on the segments of “not truth,” “truth-likeness,” and “verisimilitude” (Bruner, 1985, p. 97). Even though a story might not be “true” in a strict sense (like a fable or a parable), it still can be charmingly truthful and credible (Bruner, 1985, p. 113)—the criteria for narrative thinking is whether something is lifelike or has a real-life sense (Bruner, 1986, p. 11). From the cognitive neuroscience viewpoint, using stories and visual images may reduce the workload of memorizing, which facilitate the enhanced recall (O’Keefe and Nadel, 1998, pp. 138–139) as discussed above. Also, they make meaning to the information which enhances the retention (e.g., Craik and Lockhart, 1972; Craik, 2016; Baddeley and Hitch, 2017).

Two powerful constructivist triggers for the narrative mode of thinking are **metaphors^12^** (“X *is* Y”) and **similes** (“X is *like* Y” or “X is *as* Y”).^13^ Bruner (1976), p. 66) assumes that the surprise produced by a metaphor reveals new connections between things; metaphors are used to reorganize and understand human experiences in a new way (Bruner, 1983, p. 205). In narrative thinking, the metaphoric richness and possible contradictions are just as important as the incident to which the metaphor refers (Bruner, 1985, pp. 104–105). A related powerful trigger is the use of the **visual image** (Bruner, 1984, 1966; Bruner et al., 1966; also Baddeley (1997, p. 133). In Bruner’s second level of learning something new (the iconic representation), the knowledge is stored primarily in the form of visual images. Baddeley (1997), p. 133 ff.; see also O’Keefe and Nadel, 1998, pp. 389–390) discusses the visual mnemonics used by ancient rhetoricians; they consider memory a matter of honor and shame. One of the tools for these ancient masters of memorization was the “memory palace” where they stored multiple pieces of information in a visual form and they were able to recall those by wandering in that virtual palace (Yates, 1966).

Another powerful trigger within the narrative thinking is a **narrative** or **story**—and especially a logically **plotted story** (Bruner, 1986, p. 39). With a plot, that is, by a logical connection of events, it is possible to create a temporal synthesis of actions, goals, and intentions in the story. The plot amalgamates the complexity of the events and creates a coherent story. A good story is open to different interpretations because it leaves things slightly vague—different listeners or readers would fill in the gaps with their own experiences and knowledge (Bruner, 1990a, p. 53). When the story is not true in the factual sense, it can be a **fable**, **parable** or **allegory**. These are related to metaphors: the non-real stories are actually a set of linked metaphors. The hidden metaphoric nature of the parables can also be **explained**. Though not always understanding the complete layers of the stories, in any event, all listeners, from children to adults, may get something from the stories or parables depending on their intellectual capacity and experiences.

A third, commonly used mnemonic trigger in narratives, not rising strictly from Bruner’s ideas though, is to combine three things together, **triple repetitions** (e.g., Schultz, 2017, p. 8). Sometimes the triple repetitions can be intensified by adding a **gradual increase or decrease** in the numbers, values, or some other features as in a famous teaching of Jesus related to the “Matthew effect” where the servants had five, two, and one talent(s).

All in all, the relevance of the narrative triggers from the contemporary thinking of memory viewpoint is that, from the cognitive neuroscience viewpoint, using stories and visual images may reduce the workload of memorizing, which facilitate the enhanced recall (O’Keefe and Nadel, 1998, pp. 138–139) as discussed above. These triggers also make *meaning* to the stimulus (see Craik and Lockhart, 1972; Baddeley and Hitch, 2017; Craik, 2017). Foremost, using narratives and stories may be strictly connected with the essential procedures of the long-term memory, namely, with our declarative memory, more specifically, with the episodic or narrative memory (see Schacter et al., 2007; Squire, 2009; Eysenck and Keane, 2010; Madore et al., 2017).

#### Bruner and Logical–Scientific Mode of Thinking—Logical Mnemonic Triggers

In the logical–scientific mode, we try to explain the physical reality with the tools of logic, mathematics, and sciences, for example (Bruner, 1996, p. 39), and, hence, we construct realities (Bruner, 1983, p. 164; Bruner, 1986, p. 114). The logical–scientific mode is based on the formal and functional structures of thinking; it is based on empirical evidence and logical proofs. Logical–scientific mode comes very close to the concept of *logos* in Aristotelian rhetoric (see Section “Bruner, Aristotelian Rhetoric and Mnemonic Triggers”). Four types of arguments can easily be differentiated: a light argument, conclusion, reference to the something incontrovertible such as hard-fact data, and logical reasoning. A **light argument** comes with triggers “because” or “for” (“X is A *because of* Y”). A **light conclusion** can be drawn with such triggers as “then,” “thus,” “hence” or “so” (“*Hence*, X is Y”). These triggers have been handled already in Section “Building on What Is Already Known” under the topic “constructing realities.” **Reference to hard-fact data** is common these days. Referencing to a published journal article or to a set of data with large sample size is as close to a fact as can be: “it has to be true.” **Logical argument** can be presented in several ways. In modern discourse we use *deductive*, *inductive, abductive*^14^, and *statistical arguments*. Another kind of logical mnemonic trigger is **logical order** in teaching and the material. If the teaching follows a logical order, it is easier to remember.

The relevance of the logical–scientific triggers from the contemporary thinking of memory viewpoint is, foremost, that they make *meaning* to the information which enhances the retention (e.g., Craik and Lockhart, 1972; Craik, 2016; Baddeley and Hitch, 2017). The process of giving arguments and logical order or to make conclusions may also relate to the basic mode of the procedural part of the long-term memory (Squire, 2009; Eysenck and Keane, 2010) though its main function is to store something that cannot be put into words (Poldrack and Packard, 2003; Ullman, 2004; Squire, 2009). Namely, it may be possible that the *contents* of the arguments are stored in the declarative memory while the *procedure and logic* used in these triggers are stored in the procedural memory. Likewise, these could be connected to the Schacter’s ideas (Schacter et al., 2007) of constructive episodic memory as a tool for imagining the future, i.e., building scenarios of possible actions and the causal relations between actions and events.

#### Bruner, Aristotelian Rhetoric and Mnemonic Triggers

Bruner and his colleagues (Feldman et al., 1990, p. 220) connect logical thinking and narrative thinking with the classical Aristotelian rhetoric. They remind us that, in Aristotelian rhetoric, the cognitive processes of the mind are divided into two types: the emotional and the rational. Aristotle identifies in his *Rhetoric* three well-known types of rhetorical “proofs,” or modes of persuasion, that is, ways of convincing the listener: *ethos*, *pathos*, and *logos*. Of these, *ethos* and *pathos* fall in the emotional or narrative mode discussed already in Section “Bruner and Narrative Mode of Thinking—Metaphors, Similes, Narratives, and Triple Repetitions as Mnemonic Triggers” and *logos* falls in the rational or logical–scientific mode discussed in Section “Bruner and Logical–Scientific Mode of Thinking—Logical Mnemonic Triggers.” Of these three, *ethos* and *pathos* are discussed in detail here because the rhetoric viewpoint opens some additional doors to the emotional and narrative thinking of Bruner and to the mnemonic triggers. Here, such rhetoric triggers, which may be related to retention and recalling are focused on.

*Ethos* refers to the character and credibility of the speaker—how the speaker can make him- or herself believable. Aristotle broadens the original meaning of the word (of moral competence) to encompass expertise and knowledge. Though Aristotle expressly remarks that *ethos* can be achieved only by what the speaker says, it seems that, in practice, the appeal of the speaker is also based on the (known or assumed) expertise of the speaker. In the modern rhetoric, this kind of *ethos* could be reached by introducing a speaker in such a way that increases the speaker’s competence, for example, “*Doctor* John Doe” or “*specialist* Jane Doe.” Hence, such actions that are used to **increase the value** or **dignity** of the teacher, lecturer, or the teaching material, may be valid triggers for enhancing the retention: it is valuable to listen to this teaching or read this material.

We use *pathos* to alter an audience’s view by appealing to their emotions. *Pathos* appeals to the emotions by using metaphors and stories discussed in Section “Bruner and Narrative Mode of Thinking—Metaphors, Similes, Narratives, and Triple Repetitions as Mnemonic Triggers” as well as in the amplification of matters. *Pathos* can be conjured by the passion of the speaker or by the number of emotional items included in the teaching. We achieve *pathos* also by weighting an important matter, introducing peculiar or new ideas to the audience, or by a hyperbole. The stronger is the *pathos* the more is the effect. Here, the focus is in the strict rhetorical triggers within the Brunerian narrative mode in enhancing the retention. This kind of hook is a rhetorical device to attract the attention of the audience and to make them want to listen to the rest of the speech. These “hooks” can also be a series of intriguing questions or number of other devices to leave the listener wanting more. Aristotle discusses a large variety of feelings such as prejudice, compassion, and anger (Aristotle, 1926, Book 1, 1:4), jealousy (Book 1, 1:5), love and hate (Book 1, 1:7; 2:5), joy and sorrow (Book 1, 2:5), shame and shamelessness (Book 2, 6:1-2), courage (Book 1, 5:10), excitement and wonder (Book 1, 11:24, 27) or amusement, relaxation, laughter and ridiculing (Book 1, 11: 29), or terror and pity (Book 1, 14:1). Some of the triggers for these emotions are discussed below.

Some practical narrative-rhetorical mnemonic triggers related to *pathos* are to **show ones feelings** (“*Woe to that person*…”); to **evoke positive** or **negative emotions** such as empowerment, comfort, and safety or disgust; to use **humor**, **anecdotes**, **jokes**, **puns**, **satire**, or **hyperbole**; to **activate the audience** with a **rhetorical question** or by a **contact with the audience** or **direct address to the audience**; or it can manifest as **playing with words**, in **aphorisms**, and in **proverbs**. Other ways to raise the *pathos* are **attaching more** **weight to what is going to be said**, **attaching more** **weight to an important matter,** or to use **intellectually challenging ideas** like **paradoxes**, **peculiar ideas,** and **ideas beyond the common understanding**. We can easily find more these kinds of rhetorical triggers related to *pathos*.

The relevance of the rhetorical ethos and pathos triggers from the contemporary thinking of memory viewpoint is, foremost, that they activate the attention. It is hypothesized that the new associations between items, and between each item and its context, are set up in the focus of attention (Cowan, 1998) and that attention determines what will be encoded (Chun and Turk-Browne, 2007). The more attention is devoted at the time of encoding the more probably we create a memory.

### Bruner and Learning by Rhymes, Rhythm, and Music—Phonological Mnemonic Triggers

For some reason, Bruner was *not* interested in such elementary mnemonic triggers as **rhymes, rhythms,** and **music** in relation with the memory. However, a cognitive psychologist Baddeley (1997), p. 134) notes that the combination of meaning and rhyme is a very powerful device for remembering. Wallace (1994) experimentally showed that text is better recalled when it is heard as a song rather than as speech. A related interesting fact is that aphasic patients, who cannot speak with words because of a severe brain damage, may be able to *sing* the words, and patients with severe speech problem can increase their word production dramatically by singing (see Skeie et al., 2010, p. 353). These phonological triggers may be cognitive or narrative—we actually do not know exactly why the music and rhythm are effective mnemonic devices. It seems that the storage of rhymes, rhythms, song texts, and other musical elements is somewhere else than where the language-related elements are (see Cohen and Ford, 1995; Kaan and Swaab, 2002; Jeffries et al., 2003).

## Cognitive, Constructivist, and Rhetoric Mnemonic Triggers—An Outline

As a conclusion to Sections “Bruner and Cognitive–Linguistic Mnemonic Triggers,” “Bruner and Constructivist Mnemonic Triggers,” and “Bruner and Learning by Rhymes, Rhythm, and Music—Phonological Mnemonic Triggers,”, the linguistic–cognitive, constructive-, and phonological–mnemonic triggers handled are collected in Table 1. The individual triggers in Table 1 are in the order found in the course of the article—they are not in order of importance or weight. The list of triggers is, obviously, not exhaustive though many relevant categories may have been detected. Experts from different domains of science may add remarkably new triggers to the list. The list is operational and theoretical in the sense that we actually do not know how good or essential mnemonic triggers they are. However, they make sense when we think about effective teaching and its effective retention. The list should be taken as a tool to widen the scope about mnemonic tools (see literature in Lubin and Polloway, 2016) and to promote experimental studies. The categorization and the list may form a basis for a theoretical framework for the later studies.

**Table 1:** Examples of mnemonic triggers based on Bruner’s ideas.

Cognitive–linguistic mnemonic triggers	Doubles (“and,” “or”) (“X *and* Y”; “X *or* Y”)
	Strict comparison (“compare” or “the same way”) (“in the same way, X *is* Y”)
	Strict comparative (“better than”) (“X is *better* than Y”)
	Strict differentiation (“differentiate” or “separate”) (“*separate* X from Y!”)
	Expression for opposite (“but”) (“X is [like] A *but* Y is [like] B”)
	Nominal counterparts (“body – cloth,” “receive – give”)
	Extreme counterparts (“good – bad,” “sheep – wolf”)
	Nominal ordering (“big – small”)
	Comparative ordering (“bigger – smaller”)
	Superlative ordering (“biggest – smallest”)
	Extreme values (“all,” “always,” “never,” “in the end” or “finally”)

Strict constructivist mnemonic triggers	Spiral teaching (enlarging the material in different rounds)
	Connecting teaching to something already known (“as you know…”)
	Connecting teaching to common concepts (e.g., ingredients, traditions)
	Structural repetition (repeating word or idea within the teaching)
	Repetition of the material (teaching the same matter again)
	Repeated testing of the learnt material

Narrative–constructivist mnemonic triggers	Metaphors (“X *is* Y”)
	Simile (“X is *like* Y”) or (“X is *as* Y”)
	Visual image
	Plotted story
	Narrative/parable/allegory
	Triple repetitions (“three paths to go”)
	Gradual increase or decrease (“1 – 2 – 3”, “3 – 2 – 1”)

Logical–scientific constructivist mnemonic triggers (including *logos*)	Light argument (“because” or “for”) (“X is Y *because of* A”)
	Conclusion (“then,” “thus,” “hence,” “so,” “therefore”) (“*hence*, X is Y”)
	Condition (“if,” “unless”) (“*if* X then Y” or “*unless* X (*no*) Y”)
	Reference to hard-fact data
	Logical argument (deductive, inductive, abductive, and statistical arguments)
	Logical order (in teaching and in the material)

Rhetoric–constructivist mnemonic triggers (*ethos* and *pathos*)	Raising the value or dignity (of the teacher or the study material)
	Showing emotions
	Evoking positive emotions
	Evoking negative emotions
	Humor (incl. anecdotes, jokes, puns, satire)
	Hyperbole
	Activating the audience (“see!,” “listen!,” “be aware!”)
	Rhetorical question (“isn’t it so?”)
	Direct address to the audience (“you!”)
	Collective address to the audience (“you all!”)
	Playing with words
	Aphorisms and proverbs
	Attaching more weight to an important matter (“mark my words!”; “remark!”)
	Attaching more weight to a saying (“surely, I say”)
	Paradoxical idea
	Idea beyond a common sense
	Peculiar ideas

Phonological mnemonic triggers	Rhymes
	Rhythms
	Singing

## Back to the Beginning

The article started with a practical example of a listening test with two sequences of 31 words. The latter sequence was:

Now, listen carefully because I will ask these concepts in the final examination. The terms of reliability and validity are ultimately important when referring to the trustworthiness of a psychometrical test.

What mnemonic triggers we can find in the sequence in comparison with the theoretical framework in Table 1? At least the following ones:

*Now*, is a rhetorical-constructivist trigger “Activating the audience.”

*Listen carefully*, is a rhetorical-constructivist trigger “Raising the value or dignity (of the study material).”

*Listen carefully*, is a rhetorical-constructivist trigger “Attaching more weight to an important matter.”

*Because*, is a logical-scientific constructive trigger “Light argument.”

*I will ask these concepts*, is a rhetorical-constructivist trigger “Raising the value or dignity of the study material.”

*Final*, is a Cognitive-linguistic trigger “Extreme value,”

*Examination*, is a Strict constructivist trigger “Connecting matter to something already known,”

*Reliability and validity*, is a Cognitive-linguistic trigger “Nominal counterparts,”

*Reliability and validity*, is a Cognitive-linguistic trigger “Doubles,”

*Ultimately important*, is a Cognitive-linguistic trigger “Extreme value,”

*Ultimately important*, is a Rhetorical-constructivist trigger “Raising the value or dignity (of the study material),”

*Trustworthiness*, is a Strict constructivist trigger “connecting matter to something already known,”

*The whole sequence*, is a Logical-scientific constructive trigger “Logical order (of the study material),”

Hence, it seems that we could find, at least, 13 mnemonic triggers in the short sequence of words for the listener to recall the sequence. Instead of repeating the words—as meaningful as they are—without the meaning, the mnemonic triggers make the learning practically effortless and effective.

## Discussion

All of us have experienced teaching sessions and educational talks of we do not remember anything but the entry and the exit of the speaker—if even that. On the other hand, all of us have experienced lectures, educational sessions, or presentations that *were* inspiring, empowering, and moving; we came across new ideas, we remember some phrases and stories—we may even be able to repeat some segments word for word from what was said in the speech. Certainly, a good teacher either has been trained in or uses natural rhetorical methods whereas a poorer one stumbles even with simple matters and basic things. A good teaching talk touches our feelings—either positive or negative ones—whereas a dull one consists mainly of semi important matters served in a way that is as dry as dust. A good presentation challenges us intellectually and emotionally whereas a dull and meaningless presentation makes no impact on our mind and feelings. Thus, we can very easily distinguish between these two extremes.

Every teacher wants to see his or her students to learn and prosper. However, *luckily* in some cases, the teacher’s effect on learning is surprisingly low. Based on the meta-analysis of 800 meta-analyses, Hattie found that the teacher effect is around 30% (Hattie, 2003, 2016; Hattie et al., 2015). That is, the teacher’s actions explain (only) 30% of the variations of the learning outcome in students and the 70% may be explained by the other factors. In many countries, Hattie’s 30% is an overrating. On the basis of PISA inquiries, Freeman and Viarengo (2014) estimate that the teacher effect is around 20% in OECD countries which impart common education to all children through the grades 1–9. In some countries, like Finland, teacher effect is around 10% (Metsämuuronen, 2017, p. 520); there is practically no differences among the Finnish schools when it comes to the pupils’ learning outcomes.^15^ Hence, teachers’ actions necessarily do not have much effect in learning if good study material is available—differences between the schools may be explained by the selection. Eventually, the learning of a learner happens in the brain of the *learner*. However, the teachers are willing do their best for the students within those limits.

Though we actually do not even know, comprehensively, what *learning* is, it has to be something that happens in the human’s brain. We do not know yet much of the exact physical location of the higher thinking at the neural level but recent research in locating the physical neural elements in sensing a location give us hint that it may be possible to find other nodes also which could be called the particles of the “mind.” When “constructing the world” in a Brunerian sense, we need particles or units for this construction. This article has focused on the tools used by an effective teacher for constructing the worlds by the teaching talk and study material. The specific focus was in the cognitive–linguistic and strict constructivist mnemonic triggers proposed to the enhance retention and retrieval of memory based on the ideas from Bruner. Some phonological triggers were touched upon too though Bruner did not seem to be interested in those.

The memory triggers serve the long-term storage and retrieval in multiple ways. First, we can connect the rhetoric-constructivist triggers to increasing the arousal and attention of the listener, as well as to building the learning situation as an emotionally and cognitively interesting, and memorable, situation supporting the construction of a strong episodic memory representation.

Retention via recollection and familiarity are known to be partly dependent on different memory systems. Recollection is more sensitive than familiarity to response speeding, division of attention, generation, semantic encoding, and requires active construction of the contents to be remembered, while stimulus familiarity is a fast, semiautomatic process (Yonelinas, 2002). Cognitive–linguistic, narrative and phonological triggers help building via categorizing (similarities and differences in the whole or details) representations that support familiarity-based retrieval. Logical-scientific triggers clearly provide food especially for recollection, giving better possibilities to build the learnt content from parts of the information without the need to remember details the learning moment or environment (episodic) or the exact fact or object (familiarity).

The mnemonic triggers found here would serve in two ways. On the one hand, to a teacher they hint what kind of cognitive–linguistic and constructivist elements could be taken into account when preparing a mnemonically rich teaching presentation. On the other hand, the results may give some ideas for an effective textbook writing to enhance understanding, retention, and retrieval of the memory of its users. In contemporary standard classrooms, the teacher does not so much “teach” in the classic sense but merely *enables* the learning process, *helping* the learners to learn. We have turned from “*being taught by*” to “*learning from*” as Biesta (2013, 2016) has described. This will give much more weight to textbooks and other study material. We can relevantly ask *how consciously, from the mnemonic viewpoint, the textbooks are prepared*. Here, this contribution of the article may be the most valuable: it has brought into light, not only the obvious rhetorical tools used by a talented teacher, but also such unconscious linguistic triggers as may naturally help the students to connect, compare, categorize or order things and to “construct worlds” in the Brunerian spirit. These triggers could be used *consciously* when preparing the teaching material.

A critical reader would have noticed that the mnemonic triggers suggested in the text have come from the heuristic grounds and they are based on a hypothesis that these kinds of triggers could be effective in teaching and learning process. This heuristic hypothesis, however, could be taken as a proposal for more rigorous studies into their real meaning in learning and retention. Mastropieri and Scruggs (1998) and Lubin and Polloway (2016) have provided us with a convincing set of studies of the most obvious mnemonic triggers. The proposed cognitive–linguistic and constructivist triggers urge new sets of experimental studies to confirm how effective they really are in retention and recalling. Intuitively, it is clear that the more sense, connections, and story we see in a piece of teaching or text the more probable it is that we would remember it.

## Author Contributions

All authors listed have made a substantial, direct and intellectual contribution to the work, and approved it for publication.

## Conflict of Interest Statement

The authors declare that the research was conducted in the absence of any commercial or financial relationships that could be construed as a potential conflict of interest.

## Footnotes

1 The results of O’Keefe (1976), O’Keefe and Dostrovsky (1971) and the studies of Moser group in Norway from 2004 on (see Rowland et al., 2016) are worth mentioning here. “They have opened new avenues for understanding other cognitive processes, such as memory, thinking and planning” as the Nobel assembly puts it in 2014 (https://www.nobelprize.org/nobel_prizes/medicine/laureates/2014/press.html). O’Keefe and May-Britt Moser and Edvard I. Moser were jointly awarded the 2014 Nobel Prize for their discoveries of cells that constitute a positioning system in the brain. This means that, for the first time, we were able to detect and locate the higher-level thinking processes at a neural level. It is still a long way before we may locate higher cognitive operations such as analyzing, synthesizing, or evaluating (see the Bloom’s taxonomy for cognitive domain in Bloom, 1956). Our understanding how the brain is actually making and recalling the memory is very limited (Epstein, 2016)—we tend to use metaphors such as “computing” or “information processing” or “memory” which all seem to be just vague images of what actually happens in the brains.

2 The term “mnemonic” (from Greek *mn*ç*moneuein*, “to remember”) tool refers to all conscious and unconscious techniques and methods a *speaker* or a *teacher* uses to enhance the retention and recall of the message in the audience. It should not be confused with “mnemonics” which are used by the *listener* or *student* as methods for remembering the information that is otherwise difficult to recall (see Bafile, 2005). Baddeley (1997, pp. 133–134) does not make the difference but the distinction serves the idea of the article.

3 e.g., Phillips (1995, 1997, 1999), Puolimatka (1999, 2002, 2003), and Nodding (2016).

4 According to Puolimatka (2003), for example, a challenge in constructivism is that the constructivist view of knowledge does not provide us an adequate epistemological framework for the *critical thinking*.

5 Spaced repetition is a learning technique that incorporates increasing the intervals of time between subsequent reviews of previously learned material (Baddeley, 1997). This comes close Bruner’s idea of spiral curriculum (Bruner, 1960): the previously learned material is taught/studied again after some time with deepened contents.

6 Tomic and Kingma (1996) nicely handle the connection of these pioneers.

7 Bruner calls the cognitive psychology as the “poor cousin” of cognitive sciences (Bruner and Goodnow, 1986).

8 Google Scholar Citation indexing system (http://scholar.google.com/citations?user=z2mOtmgAAAAJ&hl=en) shows that this book has been cited more than 17,000 times at the time of finalizing the article (November 18, 2018).

9 Haggbloom et al.’s (2002) statistics were based on articles published in *psychological* journals and introductory *psychology* textbooks. In these sources, Bruner was cited 3,279 times at the time of their article. However, Bruner is cited much more often in *other* fields. According to Google Scholar Citation indexing system (http://scholar.google.com/citations?user=z2mOtmgAAAAJ&hl=en), Bruner has been cited more than 222,000 times (November 18, 2018). His four books *Acts of Meaning*, *Actual Minds Possible worlds*, *The Process of Education*, and *Toward a theory of instruction* alone are cited more than 61,000 times.

10 Note the difference between *metaphor* (“X *is* a fox”) and *simile* (“X is *like* a fox”).

11 It may be worth noting the difference between *repetition* as a student’s activity and (*structural*) *repetition* as a teacher’s activity. As a student activity, repetition is not a mnemonic device or trigger in the same sense as discussed in this article.

12 Though Bruner is used here as the main source, cognitive linguistic Lakoff’s remarkable work on the use of metaphors (Lakoff, 1993; Lakoff and Johnson, 1980) is also worth exploring.

13 There are some other relevant categories of figurative language than just metaphors and similes (see Glucksberg, 2001, p. 141; Jaszczolt and Turner, 2003, p. 141; Montgomery et al., 2007, pp. 118–121; Harley, 2014, p. 337). In a **metaphor**, “he/she *is* a fox” and in **simile** “he/she is *like* a fox.” In **metonymy**, “the *fox* sent an email” while referring to a foxy boss. In **synecdoche**, “the foxes are coming” when referring to all the different animals in the lot. An **allegory** or **parable** is a set of many metaphors: “the fox dam gathers her cubes in the evening” as an image of how a human mother would take care of her child. An **apostrophe** is used especially in poetry as a rhetorical device when personifying someone or something who is, actually, not there, like “Woe to *yee*, the foxes!” **Idioms** are “frozen metaphors” as in “fox’s nest”’ or “lion’s den”). **Fables** are animal stories where the animals represent humans and have human characteristics: “Once, a fox and a bear discussed with each other.” **Symbols** are things that stand for something else more abstract like in “a fox” as a symbol for a wit or sly person. **Hyperbole** is an overstatement not intended to be taken literally is also figurative language. A **pun** is as an expression of intended for a humorous effect by exploiting different meaning in words: “The bear wondered why the fox took the stick. Then it hit him.”

14 Abductive reasoning is used when, based on an interpretation of collected data, a researcher assembles or discovers such combinations of features for which there are no appropriate explanation or rule in the store of knowledge that already exists (e.g., Reichertz, 2007, p. 219).

15 The “teacher effect” or “school effect” is higher in the countries where there are wide differences between the student performance *and* where the private schools select the highest performing portion of the students. In those cases, the differences between the schools are high, variation within the schools is low, and, hence, the mathematical procedure used (multilevel modeling) detects that the “school” or “teacher” explains the difference highly (see Metsämuuronen, 2017, p. 520). In Nepal, for example, the “school effect” is 68% (*ibid*. p. 520). In many cases, the differences between schools can be explained by the selection of the students and in lesser extent by teachers’ actions. We have very few large-scale experimental studies that would tell what the teacher’s effect in learning really is.
